# Practical Thoughts

**Published:** 1863-05

**Authors:** Wm. A. Pease


### PRACTICAL THOUGHTS.

BY DR. WM. A. PEASE.

(Suggested by the dissolution of the Mad River Valley Association.)

The Mad River Valley Dental Society has ceased to exist. As a directing and controlling body, that exercised a supervisory care over all its members—sustained the weak, restrained the lawless or refractory, counselled the inexperienced, and wore off the sharp edges of a too active rivalry—it is now no more. Each of us is now subject only to the law of that common manhood which nature or culture has given him, and the restraint that society has thrown around him. We are no longer affected by, or responsible for, the actions of each other; we are individual, isolated, co-workers it may be in the same cause, but pursuing our labors according to the lights, the interests, or the passions of the moment. We have met together often; we shall meet now no more. Ours was a good society. It had some earnest, able, cultivated men—men of high purpose and generous and noble impulses. They formed a good nucleus for it, or any other society—a strong, healthy, muscular heart. Although it had often symptoms of pericarditis, there were never any of carditis; no rough sounds, no friction, no inflammation; all was healthy and normal. But its heart, though healthy and strong, was unable to vivify and nourish all of its members, and it died—died of atrophy.

"I come not to praise Cæsar, but to bury him."

There is a maxim that, "out of death comes life." Perhaps some of us may be individually benefited by this sudden demise, and also by knowing the cause of it.

Wherever any society is so organized that it is the interests of all of its members to sustain it, to labor for its advancement, and feel a pride in its success, that society will prosper, be useful, and respected by others. But when it is so organized that there is a strong pecuniary interest for some of its members to evade its provisions, or violate its rules of government, and it is of no great benefit to any of its members, it will soon begin to languish, and it will die. This was the condition of our society, and it will be the same with any society, having any considerable number of members out side of large cities, which establishes a fee bill so high that it is the interest of any of its members to disregard it. Some men can not resist temptation. The difficulties of establishing a dental society, having a fee bill and bye-laws, are greater than they are in any other profsssion, because its members have a two-fold vocation, viz: they are professional and mechanical. For their professional labors the fee bill has never been sufficiently high to afford an adequate compensation for their eduation and skill; and, on the contrary, it has always been too high for the skill necessary to make a practical set of teeth. Hence, there has always been a great temptation to disregard or evade it in all places where there are members not thoroughly established in business, or having inferior skill; and this was greatly increased in those places where many practitioners did not belong to the society. The fee bill for professional services has nowhere been so high that a man could charge less and be honest to his patient; but for the manufacture of artificial dentures it has always been too high for the education, apprenticeship and skill necessary to make a set of teeth. It is altogether higher than the same amount of skill receives in other mechanical pursuits; and there is no good reason why a dental mechanic should receive a compensation so out of proportion to that received in other branches of mechanics, and the community have a just right to demand that they should receive dental wares at a greatly reduced price. I know that a man may bring to the manufacture of a set of teeth so high a skill that it shall become a work of art, and be worthy of all he may receive for it; but for practical bread and butter eating machines, as they are ordinarily made, the price is too high. This is recognized by the community, and it is inferred that the price for saving a tooth is equally high. As the skill necessary to make a set of artificial teeth is no greater than is possessed by ordinary mechanics, the high reward for it is filling the land with artizans, who attempt to operate upon the mouth, and in this work great injury to dentistry and the community. Essentially mechanics, they obey the laws of trade, and advertise and solicit business in the usual manner of trade's people. They become peddlers, mendicants, who go from village to village, and from door to door, in pursuit of business for the sale of their wares. As mechanics this might be tolerated, although the generality of mechanics choose rather to be sought, like a woman, than to seek for business away from the shop. But for dentists, professional men, this course is degrading and disgusting, and condemned by all other professions. What community would allow lawyers to foment difficulties and law suits out of every village or street bickering? What man would allow a doctor to approach his wife and tender his services as an accoucheur, much less expatiate on his method of practice and skill? and yet this is little more intolerable and disgusting than for a dentist to go from door to door soliciting business.

All women wish to be "as beautiful as they can." They may know that their teeth are defective, and try to hide it, and even fancy that it is little observable, and no great detriment to their personal appearance. They neglect to have their teeth filled, sometimes from more pressing demands for their money, sometimes from doubts of the value of fillings, and sometimes from dread of pain, which is often due to the bungling and unskillfulness of the operator. They or their friends have employed mechanics, and are disgusted with dentistry; so they neglect their teeth.

The commonest decency will generally restrain a man from noticing or commenting on the defects or malformations of another in his presence, and especially is this true of women. But these self-styled dentists find one of their chief sources of profit in shaming women into patronage—shaming them to sacrifice teeth that are really valuable, that can readily be saved, for the purpose of getting artificial ones, which can never be as useful, and generally, as manufactured by them, are not as beautiful. They approach a woman with confidence and effrontery; they magnify the defects of her teeth, expatiate on their damaging effect on her personal appearance and beauty, perhaps coarsely compliment her in other respects, until her blushes and mortified air show they have wounded her sensibility and pride, when they redouble their efforts, as they then know she will be an easy victim.

It is for dentists to say whether these practices are longer to continue—whether, as protectors of the community from quackery and mal-practice, as educators of the people upon dental subjects, they are consistent with their duty to themselves, their profession and the community. The change of practice will cost an effort, perhaps a sacrifice. It will conflict with the ideas many have hitherto held of dental dignity, but it must come, for they cannot be avoided so long as the price of artificial dentures is as high as at present. The mechanics must be starved out by a material reduction of the price of artificial sets of teeth, or else they will starve out dentists in all places outside of large cities, and materially lessen their usefulness there. They can make a set of teeth for the upper jaw on rubber for thirty or thirty-five dollars, a slight reduction of the price charged by dentists, and live on the profit a month. To them societies and high fixed fee bills are a God send. They thrive and multiply under them, and flock to all places, where they are like vultures to a battle field. Why should they not? It requires a short apprenticeship, little skill and capital to make them; it gives them position as doctors, in which they can work little and loaf much—it is a deal easier than the lot of other mechanics, who earn their two dollars a day at hard labor. There is no good reason for this. There is no need for half the dental offices there are; the community do not require it; dentistry does not require it, and it is unjust to force people to pay exorbitant prices for wares that can be better and profitably made at a manufactory at less than half the present prices.

The price must and will come down; it is already down in the East, let it come quickly down in the West. Let dentists control the movement, open a manufactory, employ or instruct suitable workmen, and control the matter. By this means dentists can educate the people; they can learn them a proper appreciation of the natural teeth; that they can generally be saved; that the utility and necessity for artificial teeth has been greatly overrated; and in this way, and in this way only, make it possible for dentistry to exist as a profession.

It is a maxim in all professions that a man should receive his highest fees for his highest skill—that which requires the most talent—has cost him the most study and labor, and is the most difficult to attain. This is peculiarly true in medicine, surgery and law, and it ought to be true in dentistry. That it is not, we have the most mortifying evidence before us. How many of the most distinguished dentists of the country derive their highest, most profitable fees from the exercise of their highest skill—that which gives them character and eminence, and is, at the same time, the most useful to the community? Probably not one. On the contrary, I think it can be safely asserted that not a single dentist receives as great a profit for his time, material and skill for saving the teeth as he would for inserting a like number of artificial ones. This is all wrong, because it is demanding from the community a higher consideration than the wares are justly worth. It is demoralizing, inasmuch as it is a constant temptation to discharge less than his duty to his patient, in order that he may derive another and a higher fee from his unfaithfulness, in the price of an artificial set of teeth. And it is suicidal, because it stimulates him the wrong way—to be content with a lower order of skill—to be less useful to the community; and it is opposed to all progress or improvement, because it will not *pay* to attempt any thing difficult or untried. In illustration of this, take the fee bill recently adopted in Cincinnati as being rather more favorable to dentists than fee bills of societies in general.

What dentist would not prefer, as affording a higher profit on his skill, time and material consumed, to make a set of fourteen teeth for sixty-five dollars, the minimum, than to insert fourteen fillings of the size and quality that will be inserted for three dollars each? And yet, sixty-five dollars is a common price for a set of teeth for either the upper or under jaw, and it will be readily paid; nay more, it will be oftner and by more persons materially increased than will be the price of filling above the minimum. And yet the people will buy the set of teeth without question, but they will grumble much at the extortionate price of saving their own teeth. Such has been their education, and such it will continue to be so long as mechanics largely outnumber dentists; so long as they solicit business from door to door, praising their wares and urging the sale of them, like shop-keepers; and so long as they advertise as they now do in the public journals.

When dentists shall have made the manufacture of artificial sets of teeth a trade, by a material reduction of the price, people will begin to see in what dentistry consists. They naturally praise that which costs them the most, and they think that in their estimation of the value and profit of fillings they concur with the profession. Why do not dentists charge more for their fillings if they are really more valuable to the patient and less profitable to them?

